# Neuroprotective Effects of Desert Milk Exosomes in LPS-Induced Cognitive Decline: Role of Microglial M2 Polarization and AMPK Signaling

**DOI:** 10.3390/nu18020315

**Published:** 2026-01-19

**Authors:** Yujie Li, Wei Lu, Wentao Qian, Xinyuan Liao, Pengjie Wang, Yi Wang, Wenya Jiao, Menghui Wang, Jingru Zhao, Jinhui Yang, Haina Gao, Hongliang Li

**Affiliations:** 1 School of Food and Health, Beijing Technology and Business University, Beijing 100024, China; liyujie_2001@163.com (Y.L.); 19979779160@163.com (W.L.); liaoxy202410@163.com (X.L.); 2 Mengniu Hi-Tech Dairy Products (Beijing) Co., Ltd., Beijing 101100, China; qianwentao2006@163.com; 3 Inner Mongolia Mengniu Dairy (Group) Co., Ltd., Hohhot 011500, China; wangmenghui@mengniu.cn (M.W.); zhaojingru@mengniu.cn (J.Z.); 710593491@163.com (J.Y.); 4 Key Laboratory of Precision Nutrition and Food Quality, Department of Nutrition and Health, China Agricultural University, Beijing 100083, China; wpj_nh@cau.edu.cn (P.W.); wangyi922217@126.com (Y.W.); jiaowenya0320@163.com (W.J.)

**Keywords:** milk exosomes, cognitive decline, hippocampal neuroinflammation, tauopathy, microglial polarization

## Abstract

Background/Objectives: Hippocampal neuroinflammation (HNF) is a key pathological feature in neurodegenerative disorders. Milk-derived exosomes, as bioactive extracellular vesicles, have underexplored potential in regulating brain neuroinflammatory responses. This study aimed to characterize desert milk exosomes (D-Exo) and investigate their neuroprotective and anti-neuroinflammatory effects in LPS-induced HNF mice model and an LPS-stimulated BV2 microglia. Methods: Exosomes were isolated from desert and non-desert milk (ND-Exo) for proteomic analysis. After pretreating BV2 cells with exosomes and stimulating with LPS, their inflammatory responses and polarization were assessed by RT-PCR. Balb/c mice were orally gavaged with D-Exo or 0.9% NaCl for 28 days before LPS injection. Cognitive function was assessed via behavioral tests, with microglial/astrocyte activation analyzed by immunofluorescence. Results: D-Exo exhibited superior stability and a unique proteomic profile enriched with proteins linked to neuroinflammation and blood-brain barrier (BBB) integrity, notably within the AMPK signaling pathway. In vitro, D-Exo shifted LPS-stimulated microglia from the M1 to the M2 phenotype. In vivo, it alleviated HNF and cognitive decline, reduced Aβ_1-42_ and Tau deposition, elevated BDNF and MAP2, and suppressed neuroinflammation and glial activation. Conclusions: D-Exo is enriched with specific proteins, attenuates neuroinflammation and cognitive decline by regulating microglial M1/M2 polarization and AMPK pathway, highlighting its preventive potential.

## 1. Introduction

Recent studies establish hippocampal neuroinflammation (HNF) as a key driver of memory and cognitive decline [1]. This impairment can persist for years and is observed even in young adults and students [2,3]. This process is largely driven by the dysregulated activation of glial cells, including microglia and astrocytes [4,5]. Following activation, these cells produce a cascade of inflammatory factors, cytokines, and oxidative substances that contribute directly to neuronal dysfunction [6].

Lipopolysaccharide (LPS) induced HNF leads to deficits in neuronal synaptic plasticity and dysregulation of microglial function [7,8]. Under inflammation, amyloid-beta_1–42_ (Aβ_1–42_) accumulates and Tau protein was phosphorylated abnormally to form neurofibrillary tangles (NFTs) [9]. Tau hyperphosphorylation not only destabilizes microtubules but also promotes microglial activation and perpetuates neuroinflammation, establishing a vicious cycle that contributes to disease progression [10]. Conversely, brain-derived neurotrophic factor (BDNF) and microtubule-associated protein 2 (MAP2) are key regulators of hippocampal neuronal survival, synaptogenesis, dendritic branching, and neurotransmitter profiles, processes that collectively enhance cognitive function [11,12]. Therefore, inhibiting Tau hyperphosphorylation and neuroinflammation may be a promising preventive strategy against HNF.

As the primary immune cells of the central nervous system, microglia play a central role in neuroinflammation [13]. Under inflammatory stimuli like peripheral inflammation crossing a compromised blood-brain barrier, microglia predominantly polarize into a pro-inflammatory M1 state [14]. Upon activation, cells not only secrete cytokines like tumor necrosis factor-α (TNF-α), interleukin-33 (IL-33) and interleukin-1β (IL-1β), but also upregulate the production of inducible NO synthase (iNOS) [15,16]. Together, they intensify inflammatory damage and neuronal loss, linking directly to cognitive decline. Conversely, promoting their transition to an anti-inflammatory M2 phenotype, which clears cellular debris under healthy conditions, has been shown to alleviate HNF and exert neuroprotective effects [17].

Food-derived exosomes function as highly stable bioactive constituents with considerable resistance to gastric acid and the ability to cross the blood-brain barrier (BBB) [18,19]. Leveraging these properties, human milk exosomes by targeting the NF-κB and p38 MAPK pathways, which mitigates LPS-stimulated microglial activation and associated neuroinflammation [20,21]. *BL99*-derived vesicles attenuate neuroinflammation primarily through the IL-33/FoxO6/P53 axis, resulting in reduced neuronal inflammation and improved synaptic integrity [22]. *Akkermansia muciniphila* extracellular vesicles alleviate gut inflammation and related cognitive impairment via multi-target actions on the gut-brain axis [23]. Allium tuberosum exosomes alleviated LPS-induced microglial inflammation by modulating gene expression, demonstrating intrinsic therapeutic potential against neuroinflammation [24]. These observations suggest that exosomes may be a prevention strategy for HNF.

The environments influence the composition of ruminant milk, promoting he enrichment of functional proteins [25,26,27]. As an example, yak milk exosomes alleviate intestinal inflammation by upregulating CD46 protein and activating the PI3K/AKT/C3 signaling pathway [28]. Camel milk from desert regions is not only rich in bioactive substances like lactoferrin but also yields exosomes with definitive anti-inflammatory properties, underscoring the functional impact of its unique provenance [29,30]. These findings indicate that changes in milk functionality are likely linked to its environment. However, the effects of milk-derived exosomes, particularly those from unique environments like desert milk, on HNF and its associated pathology remain poorly defined. This study demonstrates that desert milk exosomes (D-Exo) possess superior stability and a unique proteomic profile compared to non-desert milk exosomes (ND-Exo). D-Exo is specifically enriched with proteins (e.g., SIL1, FN1, FGG) linked to the regulation of HNF and the AMPK signaling pathway. Through this pathway, D-Exo attenuates HNF by modulating microglial polarization, thereby reducing neuroinflammatory responses and oxidative damage. This ultimately alleviates cognitive decline, pathological Tau phosphorylation, and Aβ_1-42_ accumulation. Our findings provide novel insights into the mechanisms by which D-Exo attenuate neuroinflammation and tau pathology, supporting their potential prevention application in HNF and neurodegenerative diseases.

## 2. Materials and Methods

### 2.1. Preparation and Analysis of Milk Exosomes

Milk samples were collected from two distinct cow colonies located at the following ranches in China: Desert milk from Shengmu Beidou Ranch in Dengkou County, Bayannur City, Inner Mongolia and Non-desert milk from Yinchuan Ranch in Ningxia City. Where husbandry practices were identical. The sole variable was the geographic and climatic environment. Following collection, milk samples were placed at −20 °C. Laboratory transfer was followed by preservation at −80 °C for long-term storage until use.

**Figure 1:**
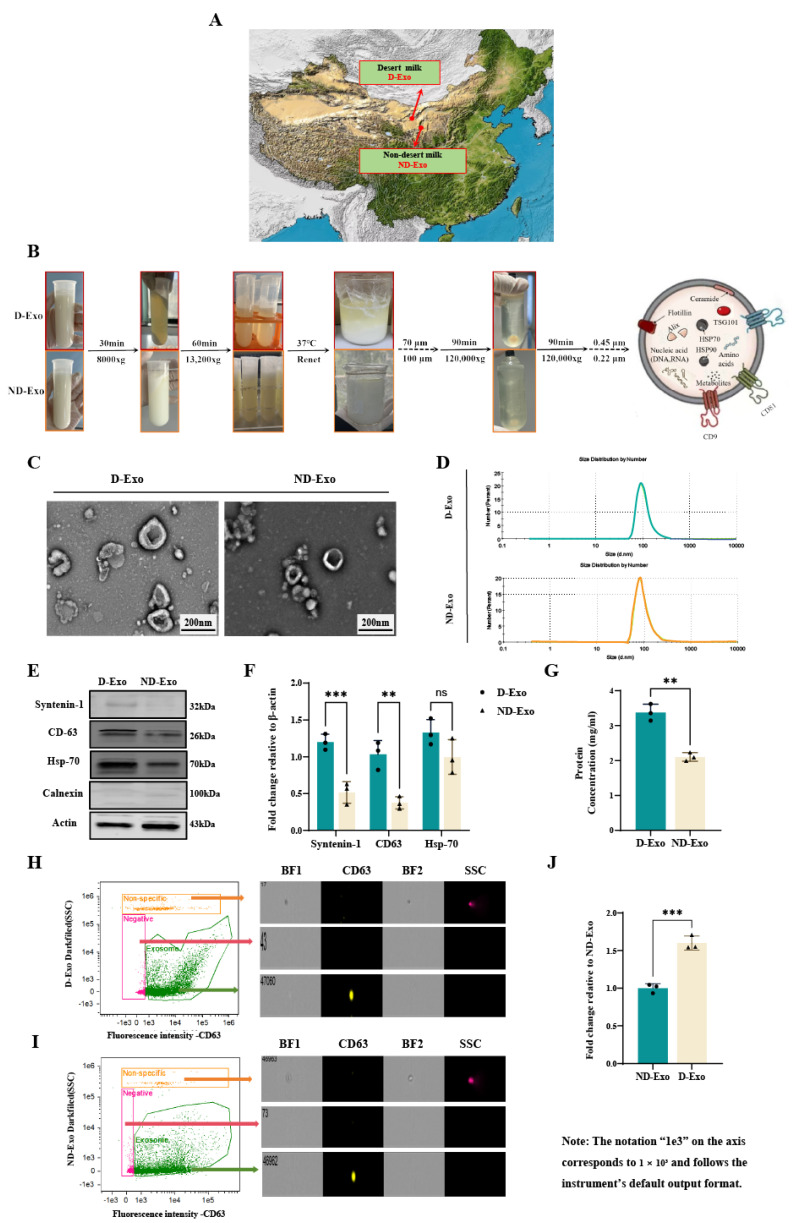
Characterization of optimized milk exosomes. (**A**) The geographical locations of the sampling sites. (**B**) Experimental protocol. (**C**) TEM images showing D-Exo and ND-Exo (scale bar: 200 nm). (**D**) NTA curve of D-Exo and ND-Exo. (**E**) Expression of characterized proteins in D-Exo and ND-Exo by WB method. (**F**) The Syntenin-1, CD63, HSP70 and Calnexin protein with representative image by WB. (**G**) The protein concentration of exosomes by BCA. (**H**) Images showing D-Exo. (**I**) Images showing ND-Exo. (**J**) The relative number of exosomes from different milk. Data are presented as mean ± SEM (*n* = 3 per group). Statistically significant differences were indicated: * *p* < 0.05, ** *p* < 0.01, *** *p* < 0.001, ns > 0.05.

Raw milk samples from each farm were pooled into 3 fractions, and isolation of milk exosomes was performed using the same method as described previously (Figure 1B) [31]. Milk exosomes were extracted through sequential centrifugation at 8000× *g* (30 min, 4 °C) and 13,200× *g* (1 h, 4 °C), followed by casein precipitation with rennet (0.025 g/L, 37 °C, 6 h). The supernatant was then subjected to ultracentrifugation (120,000× *g*, 90 min, 4 °C; Type 70 Ti rotor, Beckman Coulter, Brea, CA, USA). Finally, the collected material was filtered through 0.45-μm and 0.22-μm membranes and resuspended in PBS.

Bicinchoninic acid assay kits (BCA; cat. #PC0020, Solarbio, Beijing, China) was quantified the protein content of isolated milk exosomes. Transmission electron microscopy (TEM, 100 kV; JEM-1200EX, JEOL Ltd., Tokyo, Japan) and nanoparticle tracking analysis (NTA; Nano ZS ZEN3600, Malvern Panalytical, Malvern, UK) were used to characterize milk exosomes. Flow Cytometry (FC) was characterized and quantificated exosomes Western blot analysis (WB) was performed to detect milk exosomes membrane marker using the following primary antibodies: Syntenin-1 (1:800; cat. #22399-1-AP, Proteintech, Shanghai, China), CD63 (1:1000; cat. #28283-1-AP, Proteintech, Shanghai, China), HSP70 (1:1000; cat. #10995-1-AP, Proteintech, Shanghai, China), along with the negative control Calnexin (1:2000; cat. #10427-2-AP, Proteintech, Shanghai, China) and loading control β-actin (1:5000; cat. #66009-1-IG, Proteintech, Shanghai, China).

### 2.2. Resistence of Milk Exosomes to Simulated Gastric Fluid (SGF)

Equal amounts of milk exosomes suspended in 100 μL of PBS were incubated in 1 mL of PBS and SGF (cat. #PH1840-500mL, Feijing, Beijing, China) at 37 °C with gentle shaking. After 4 h, milk exosomes stability was assessed by measuring size and protein concentration. Milk exosomes integrity was assessed by TEM morphological analysis.

### 2.3. Proteomic Profiling via LC-MS/MS and MaxQuant Analysis

Proteins were digested following the filter-aided sample preparation (FASP) protocol [32]. Samples containing 200 µg protein were lysed in SDT buffer (4% SDS, 150 mM Tris-HCl, 100 mM DTT, pH 8.0), followed by detergent removal and buffer exchange using UA buffer (8M Urea, 150 mM Tris-HCl, pH 8.0) via 10-kDa ultrafiltration. After reduction and alkylation with 100 mM iodoacetamide, proteins were digested overnight with trypsin at 37 °C. The desalted peptides were vacuum-dried (Empore™SPE Cartridges C18; Sigma, St. Louis, MO, USA), reconstituted in 0.1% formic acid, and stored at −80 °C after concentration measurement (NanoDrop 2000, ThermoFisher, Waltham, MA, USA).

Peptide separation and mass spectrometry were performed using an Easy nLC 1200 system coupled to a Q Exactive HF mass spectrometer (Thermo Scientific, Waltham, MA, USA). Samples (1.5 µg) were loaded onto an EASY-Spray™ C18 trap column (P/N 164946, 2 cm long, 75 µm inner diameter, 3 μm resin; Thermo Scientific, USA) and separated on an analytical column (25 cm × 75 µm, 2 µm; ES802) with a 90-min gradient of buffer B (84% acetonitrile, 0.1% formic acid) at a flow rate of 250 nL/min.

Full MS scans (resolution: 60,000; range: 300–1800 m/z) were acquired, followed by data-dependent MS/MS scans (resolution: 15,000) on the top 20 ions. For proteomic analysis, MS/MS spectra were processed with MaxQuant (v.2.4.2.0) for database searching, applying a one-unique-peptide criterion for protein identification. Subsequent quantitative and statistical analyses (Benjamini-Hochberg corrected, FDR < 0.05) were carried out in Perseus (v.1.6.2.3).

### 2.4. Cell Culture and Stimulation

BV2 microglial cell (iCell, Bioscience Inc., Shanghai, China) line was cultured in DMEM (cat. #11965092, Gibco, Grand Island, NY, USA) supplemented with 10% FBS (cat. #SX1101, SORFA, Huzhou, China) and 1% penicillin-streptomycin (P/S) (cat. #15140122, Gibco, USA) at 37 °C under 5% CO_2_. For subculturing, confluent cells were detached using 0.25% trypsin-0.02% EDTA (cat. #C0201, Beyotime Institute of Biotechnology, Shanghai, China). For phenotypic polarization, the M1 phenotype was induced by 6-h LPS (100 ng/mL; cat. #L2880-100MG, Sigma, USA) stimulation, while the M2 phenotype was induced by 24-h IL-4 (20 ng/mL; cat. #PHC0044, Invitrogen, Carlsbad, CA, USA) treatment.

### 2.5. Cell Treatment

Cell viability was evaluated using a CCK-8 assay (cat. #MA0218, Meilunbio, CHN). BV2 cells were seeded in 96-well plates, serum-starved, and divided into 6 treatment groups: control group, LPS group (stimulated with 1 μg/mL LPS), D-Exo (200, 400 ng/mL) + LPS group and ND-Exo (200, 400 ng/mL) + LPS group (n = 6). The experimental timeline is illustrated in Figure 5A. After treatments, CCK-8 reagent was added, followed by incubation and measurement of absorbance at 450 nm.

Intracellular reactive oxygen species (ROS) levels were quantified using a DCFH-DA assay (cat. #S0033S, Beyotime Institute of Biotechnology, Shanghai, China). Following treatments, cells were incubated with DCFH-DA, and the resulting fluorescence intensity was measured to determine ROS production.

### 2.6. Animal Grouping and Treatment

The study was conducted in accordance with guidelines approved by the Animal Experimentation Ethics Committee of China Agricultural University (AW71705202-6-03). A total of 24 male Balb/c mice (6 weeks old, 20–22 g), sourced from Beijing Huafukang Biotechnology. Using a random number table, animals were randomly allocated into three experimental groups: Control, LPS and D-Exo (7 mg/kg per day per mice) group. D-Exo group was orally gavaged with 28 days of the study period, Control and LPS group mice were administered a 0.9% NaCl solution. In 29 Days, D-Exo and LPS group with LPS treatment (1 mg/kg) (cat. #L2880-100MG; Sigma, USA) for 6 h, while control mice were administered an equal volume of saline (Figure 7A). The body weight of mice in each group was recorded daily.

### 2.7. WB

Hippocampal tissue were homogenized in RIPA buffer, and the supernatant was collected for protein quantification with a BCA kit (cat. # PC0020, Solarbio, Beijing, China). After denaturation in loading buffer at 95 °C for 10 min, proteins were resolved by 10% SDS-PAGE and subsequently transferred onto PVDF membranes. Following blocking with 5% BSA, the membranes were incubated overnight at 4 °C with the primary antibody for FN1 (cat. #15613-1-AP, Proteintech, Shanghai, China), SIL1 (cat. #24110-1-AP, Proteintech, Shanghai, China), AMPK (cat. #10929-2-AP, Proteintech, Shanghai, China), phospho-AMPK (cat. #80209-6-RR, Proteintech, Shanghai, China), and GAPDH (cat. #10494-1-AP, Proteintech, Shanghai, China). Following incubation with an HRP-conjugated secondary antibody, protein bands were detected using a chemiluminescence imaging system. Band intensity was analyzed with Image J 2x 2.1.4.7 software.

### 2.8. Morris Water Maze (MWM)

Spatial learning and memory were tested using the MWM. The apparatus was a circular pool (120 cm diameter, 25 °C) containing a hidden platform (10 cm diameter) submerged in quadrant III. During training, mice were allowed 60 s to find the platform. In the subsequent probe trial (platform removed), trajectories, platform crossings, and the time/distance spent in the target quadrant were recorded over 60 s.

### 2.9. Novel Object Recognition Experiment (NOR)

Recognition memorynin mice were evaluated using the NOR. During the familiarization phase, mice freely explored an arena containing two identical objects (A and B) for 5 min. After a 24-h interval, one familiar object (B) was replaced with a novel object (C) for the test phase. Mice were reintroduced to the arena for another 5 min session, and their exploratory interactions with both objects were recorded. The time spent investigating the familiar object (TA) and the novel object (TC) was quantified. A recognition index was calculated as [TC/(TA + TC)] × 100% to evaluate memory performance.

### 2.10. Histopathological Examinations

Following perfusion, brain tissues were fixed in 4% paraformaldehyde solution (cat. # BL539A, Biosharp, Hefei, China), dehydrated, paraffin-embedded, and sectioned at 3 μm thickness. Sections were stained with hematoxylin and eosin (HE). Neuronal damage in the hippocampal CA1 region was assessed by quantifying surviving neurons and eosinophilic cells across three random microscopic fields per section using Image J2x 2.1.4.7 software.

Hippocampal sections were subjected to immunohistochemical (IHC) staining to assess the expression of Aβ_1−42_ (cat. #A9810, Sigma-Aldrich, St. Louis, MO, USA), Tau (cat. #10274-1-AP, Proteintech, China), MAP2 (cat. #17490-1-AP, Proteintech, China) and BDNF (cat. #25699-1-AP, Proteintech, China) using specific primary antibodies. Antigen visualization was achieved with an HRP-conjugated secondary antibody (cat. #ab205718, abcam, Cambridge, UK) and DAB chromogen (cat. #k3468, Dako, Glostrup, Denmark). After microscopic examination of morphological changes, the average optical density (AOD) of positive signals in three random CA1 fields was quantified using ImageJ software.

Immunofluorescence (IF) staining was performed to assess the expression of Iba-1 (cat. #ab178846, abcam, UK) and GFAP (cat. #16825-1-AP, PTG, Mason, OH, USA) in hippocampal sections. After incubation with specific primary antibodies overnight at 4 °C, sections were treated with an Alexa Fluor^®^ 555-conjugated secondary antibody (cat. # ab150078, abcam, UK). Nuclei were counterstained with DAPI (cat. #B0025, POWERFUL BIOLOGY, Stanford, CA, USA). Following PBS washes, fluorescence signals were visualized and captured using a fluorescence microscope (Thermo Fisher, Waltham, MA, USA).

### 2.11. Enzyme-Linked Immunosorbent Assay (ELISA)

Concentrations of TNF-α (cat. #EK282, RUIXIN, Wenzhou, China), IL-33 (cat. #RX203053M, RUIXIN, Wenzhou, China), IL-1β (cat. #RX203063M, RUIXIN, Wenzhou, China) and IL-10 (cat. #RX203048M, RUIXIN) in cell supernatant and serum were quantified using ELISA kits. Blood samples were collected in EDTA-treated tubes and centrifuged (3000× *g*, 15 min, 4 °C). The resulting supernatant was collected for analysis.

### 2.12. Quantitative Real-Time Polymerase Chain Reaction (qRT-PCR)

RNA from hippocampal tissue and cultured cells was isolated following the Trizol Reagent protocol (cat. #15596026, Invitrogen, USA). RNA quality was verified via a NanoDrop 2000 spectrophotometer (Thermo, Shanghai, China). Subsequently, cDNA was synthesized with the PrimeScript RT Reagent Kit (Cat. #DRR037A, TaKaRa, Shiga, Japan). Quantitative PCR was performed using TB Green Premix Ex Taq (Cat. #RR420A, TaKaRa, Shiga, Japan) for amplification. Gene expression levels were determined by the −2^ΔΔCt^ method with normalization to GAPDH. The primer sequences used are listed in Table A1.

### 2.13. Data Processing

Data are expressed as mean ± standard error of the mean (SEM), and statistical significance was set at * *p* < 0.05. All analyses were conducted using Prism 9.2 (GraphPad Software, San Diego, CA, USA), employing one-way ANOVA followed by Tukey’s multiple comparison test.

## 3. Results

### 3.1. D-Exo Show Superior Characteristics

The geographical locations of the sampling sites are illustrated in Figure 1A. Exosomes were isolated from the milk supernatant using Figure 1B method. D-Exo and ND-Exo exhibited the characteristic cup-shaped morphology and size (50–200 nm) under TEM (Figure 1C). NTA confirmed these findings, with D-Exo showing a peak diameter of 158.5 ± 1.56 nm (poly dispersity index, PDI: 0.139) and ND-Exo 151.8 ± 6.43 nm (PDI: 0.198) (Figure 1D). WB confirmed exosomal marker enrichment and the absence of calnexin contamination. Notably, D-Exo exhibited significantly higher expression of these markers than ND-Exo. Furthermore, the protein concentration in D-Exo was significantly greater than that in ND-Exo as measured by BCA assay (*p* < 0.01; Figure 1G). FC further revealed that D-Exo preparations contained 1.5-fold more particles than ND-Exo (*p* < 0.001; Figure 1H–J). Collectively, these results indicate that D-Exo is a superior source for obtaining high-quality exosomes.

### 3.2. D-Exo Exhibits Superior Gastrointestinal Stability

**Figure 2:**
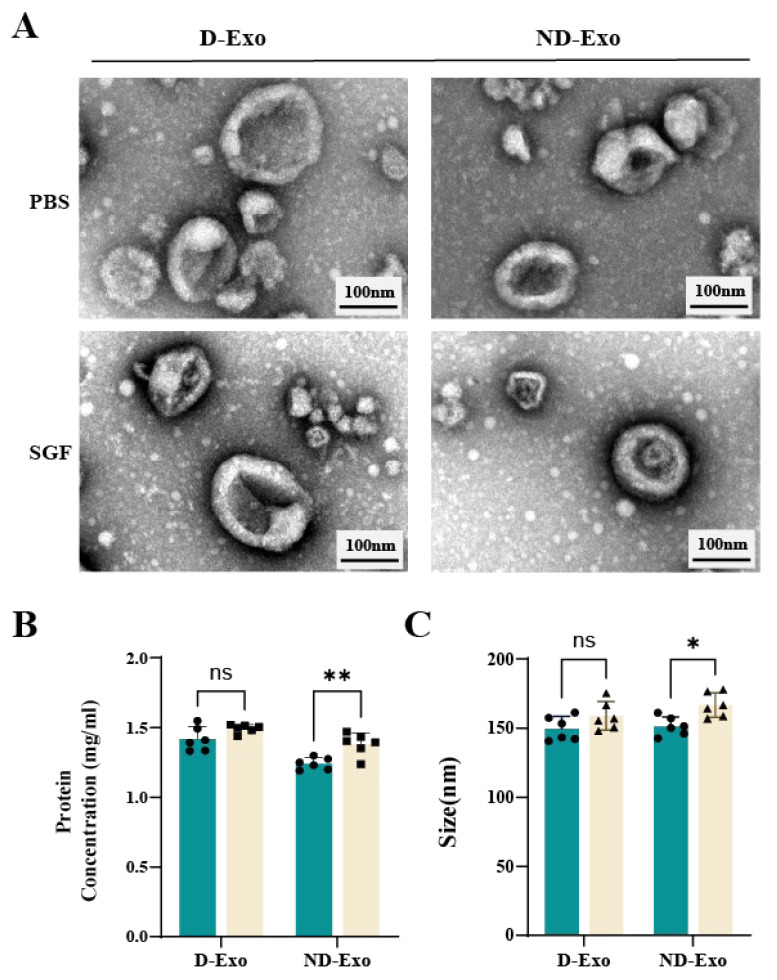
D-Exo Shows Greater SGF Stability Than ND-Exo. (**A**) TEM at 100 nm field of view compared milk exosomes morphology in PBS versus SGF-treated groups. (**B**) Plot of protein concentration before and after SGF of milk exosomes. (**C**) Plot of size before and after SGF of milk exosomes. Data are presented as mean ± SEM (*n* = 6 per group). Statistically significant differences were indicated: * *p* < 0.05, ** *p* < 0.01, *** *p* < 0.001, ns > 0.05.

Following SGF digestion, both D-Exo and ND-Exo retained their characteristic cup-shaped morphology, similar to the PBS control (Figure 2A). However, quantitative analysis revealed that D-Exo maintained stable protein concentration and particle size (*p >* 0.05), whereas these parameters changed significantly in ND-Exo (*p* < 0.05; Figure 2B,C). These findings indicate that D-Exo possesses superior gastrointestinal stability compared to ND-Exo.

### 3.3. Proteomic Analysis Reveals Enriched Proteins in D-Exo

**Figure 3:**
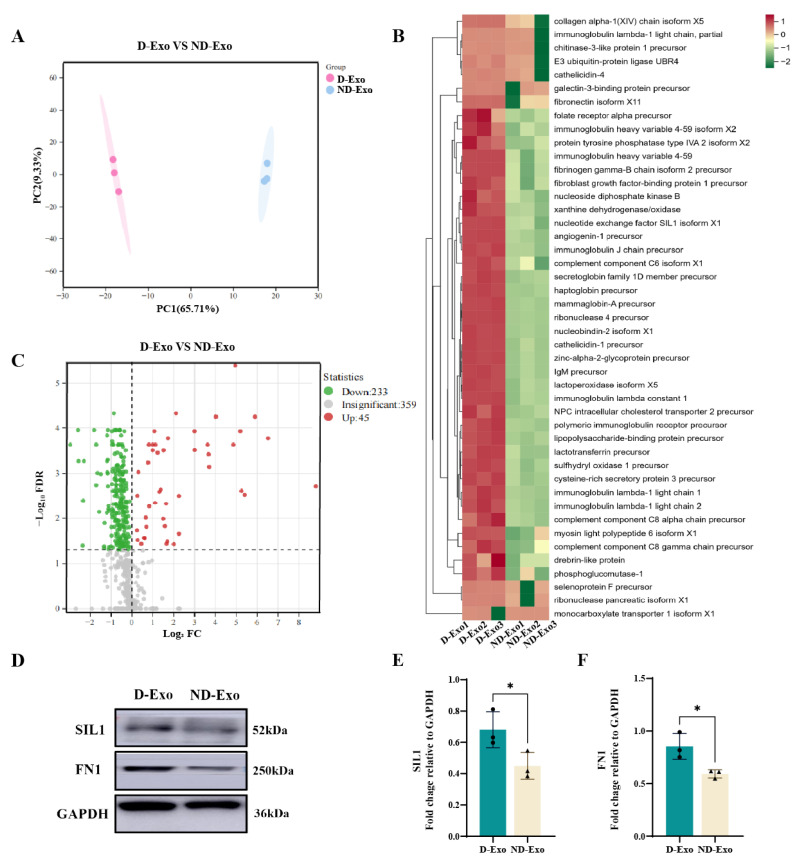
Quality assessment of proteome data by label-free quantification. (**A**) Principal component analysis. (**B**) Hierarchical clustering analysis of differential protein expression profiles between D-Exo and ND-Exo. Red = Exosomes proteins with higher expression, green = Exosomes proteins with lower expression. (**C**) Difference expression pattern between D-Exo and ND-Exo. FDR = false discovery rate; FC = fold change. (**D**) The SIL1, FN1 protein with representative image by WB. (**E**,**F**) Expression of SIL1 and FN1 proteins in D-Exo and ND-Exo by WB method. Data are presented as mean ± SEM (*n* = 3 per group). Statistically significant differences were indicated: * *p* < 0.05, ** *p* < 0.01, *** *p* < 0.001, ns > 0.05.

Proteomic analysis revealed distinct protein profiles between D-Exo and ND-Exo, with 571 protein groups identified in total. The complete dataset is publicly available in the ProteomeXchange Consortium [33,34] under accession number PXD071863. Data quality was assessed by PCA and hierarchical clustering of the MS/MS raw data. PCA clearly separated D-Exo and ND-Exo into distinct clusters (Figure 3A), and hierarchical clustering further grouped the quantified proteins into two independent branches, corresponding to each exosome type (Figure 3B). These results confirm the high reproducibility of the mass spectrometry measurements.

Comparative analysis of the proteomic profiles revealed significant differences between D-Exo and ND-Exo. LC-MS/MS quantification identified 278 milk exosome proteins common to both groups. Under the criteria of |fold change| > 1 and *p* < 0.05, 45 proteins were significantly upregulated and 233 downregulated in D-Exo relative to ND-Exo (Figure 3C). Among the differentially expressed proteins, Nucleotide exchange factor SIL1 (SIL1), Fibronectin 1 (FN1), Nucleobindin-2 (NUCB2), and Fibrinogen gamma chain (FGG) were most enriched in D-Exo (Table S1).

### 3.4. AMPK Signaling and Protein Interactome in D-Exo Mechanism

**Figure 4:**
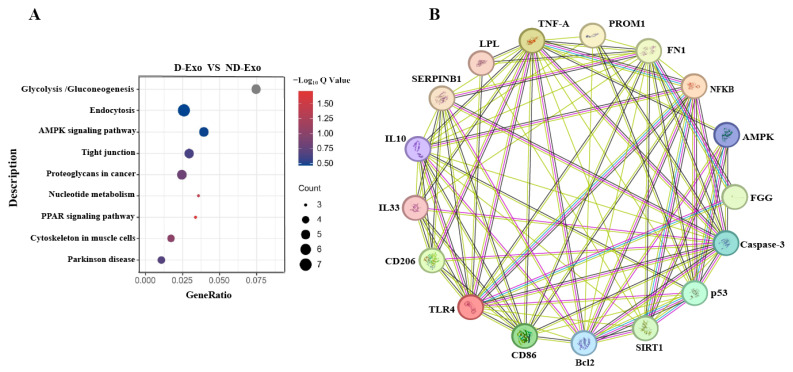
Qualitative proteome comparisons of D-Exo and ND-Exo. (**A**) KEGG pathway analysis (proteins number ≥ 3). (**B**) PPI network (enrichment *p*-value: 1.55 × 10^−12^). Data are presented as mean ± SEM (*n* = 3 per group). Statistically significant differences were indicated: * *p* < 0.05, ** *p* < 0.01, *** *p* < 0.001, ns > 0.05.

Proteomic analysis revealed differentially expressed proteins, including SIL1, FN1, NUCB2, LPL, and SERPINB1, which are associated with neuroinflammation and BBB function and enriched in the AMPK pathway (Figure 4A). To infer functional connections, a protein-protein interaction (PPI) network was generated based on medium-confidence interactions (score > 0.4). Database curation and experimental evidence confirmed a particularly strong interaction between FN1, FGG, PROM1, LPL, and SERPINB1 within this network (Figure 4B). In contrast, SIL1 and NUCB2 showed no significant connections, which may be due to its primary role as an endoplasmic reticulum chaperone, influencing neuroinflammation indirectly through proteostasis and stress response pathways.

### 3.5. Inhibitory Effect of Milk Exosomes on LPS-Induced Damage of BV2 Cells

**Figure 5:**
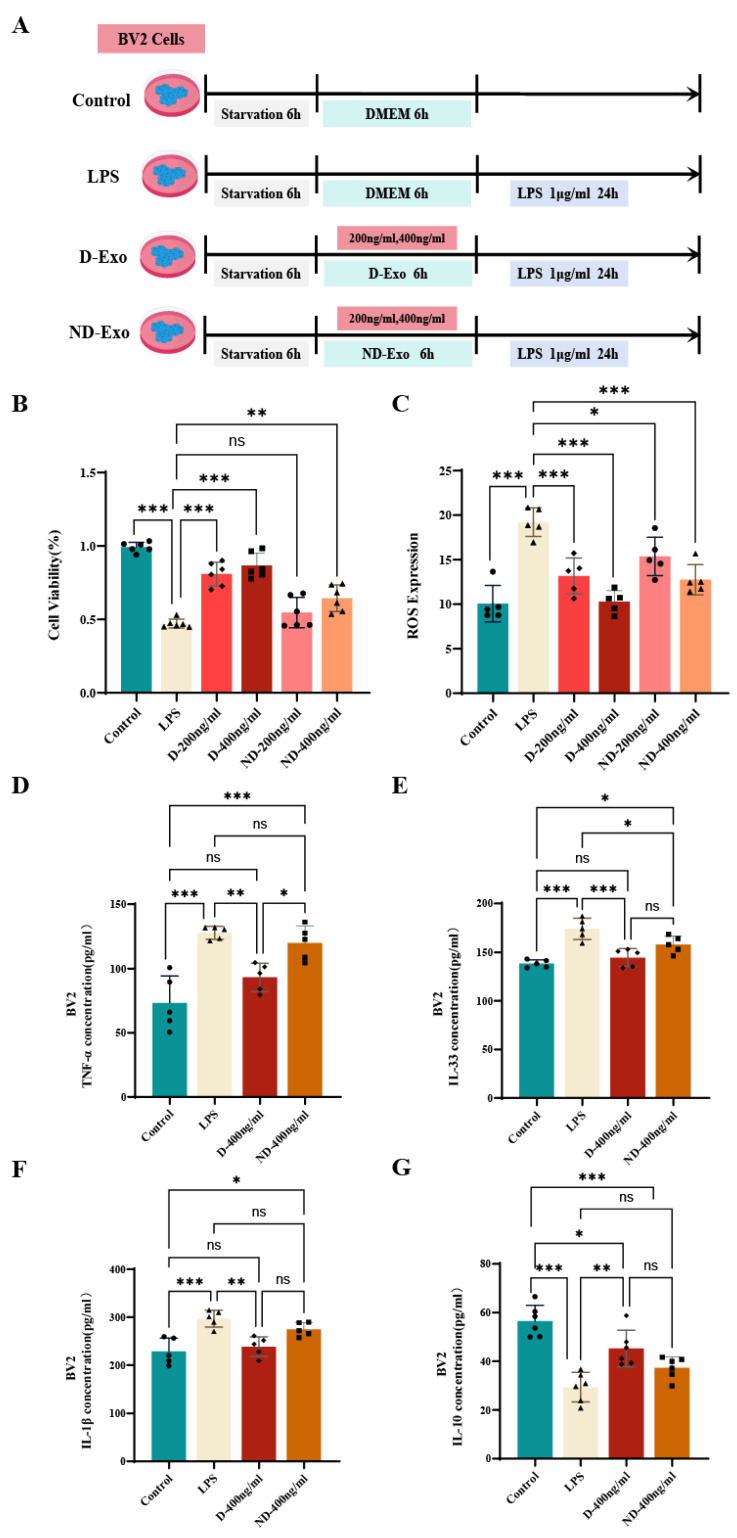
Impacts of milk exosomes on cell viability and oxidative stress level in BV2 with LPS administration. (**A**) Experimental protocol of BV2. The BV2 were classified into Control group, LPS group, D-Exo (200 ng/mL), D-Exo (400 ng/mL), ND-Exo (200 ng/mL) and ND-Exo (400 ng/mL). (**B**) Cell viability was assessed through CCK-8 (*n* = 6). (**C**) Quantitative analysis of ROS expressions based on DCF fluorescence intensity (*n* = 6). (**D**–**G**) TNF-α, IL-33, IL-1β and IL-10 in BV2 cell supernatant by ELISA (*n* = 5). Data are presented as mean ± SEM of per group. Statistically significant differences were indicated: * *p* < 0.05, ** *p* < 0.01, *** *p* < 0.001, ns > 0.05.

To evaluate neuroprotection, BV2 cells were pretreated with D-Exo or ND-Exo, respectively, followed by LPS stimulation (Figure 5A). CCK-8 assay showed that D-Exo (200 and 400 ng/mL) and ND-Exo (400 ng/mL) significantly counteracted LPS-induced cytotoxicity (*p* < 0.01), restoring viability to 80.92%, 86.63%, and 64.43%, respectively (Figure 5B). This superior bioactivity was further supported by its potent suppression of LPS-elevated ROS levels. As shown in Figure 5C, LPS stimulation markedly increased ROS production (*p* < 0.001), which was most effectively suppressed by D-Exo and ND-Exo at 400 ng/mL (*p* < 0.001), further supporting its superior bioactivity. These results demonstrate that D-Exo exhibits a dose-dependent protective and anti-inflammatory effect, with superior efficacy observed at 400 ng/mL compared to 200 ng/mL, and overall outperforms ND-Exo against LPS-induced cell damage. Furthermore, ELISA confirmed that D-Exo (400 ng/mL) effectively modulated the inflammatory response by reducing pro-inflammatory cytokines, including TNF-α (*p* < 0.01), IL-33 (*p* < 0.001), and IL-1β (*p* < 0.01) (Figure 5D–F) and increasing the anti-inflammatory cytokine IL-10 (*p* < 0.01) (Figure 5G).

Collectively, these in vitro results establish the stronger cytoprotective, antioxidant, and anti-inflammatory efficacy of D-Exo over ND-Exo, providing a rationale for its selection for in vivo studies.

### 3.6. Anti-Inflammatory Effects of Milk Exosomes in Microglia

**Figure 6:**
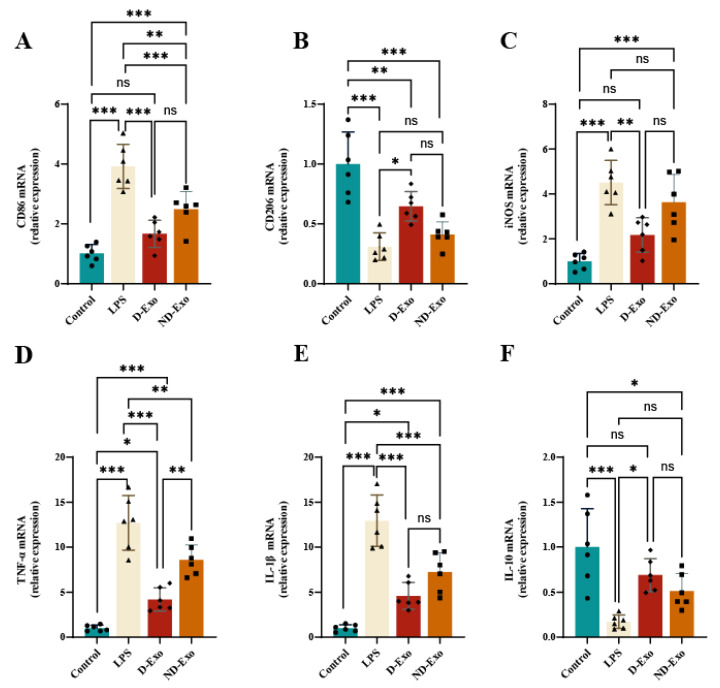
D-Exo inhibits M1 polarization and proinflammatory cytokines in LPS-induced BV2 microglia. (**A**,**B**) mRNA expression of GAPDH, CD86, and CD206 was measured using qPCR. (**C**–**F**) Changes in intracellular iNOS, TNF-α, IL-1β and IL-10 mRNA levels in LPS-stimulated BV2 cells treated with 400 ng/mL D-Exo and 400 ng/mL ND-Exo. Data are presented as mean ± SEM (*n* = 6 per group). Statistically significant differences were indicated: * *p* < 0.05, ** *p* < 0.01, *** *p* < 0.001, ns > 0.05.

Based on the prior findings, D-Exo and ND-Exo at 400 ng/mL were chosen for further investigation into their effects on microglial polarization. qRT-PCR analysis demonstrated a distinct regulatory capacity of D-Exo. It significantly downregulated the M1 marker CD86 (*p* < 0.001) while upregulating the M2 marker CD206 (*p* < 0.05) in LPS-stimulated BV2 cells, effects not observed with ND-Exo (Figure 6A,B). Further analysis of polarization-associated genes showed that D-Exo effectively suppressed the expression of iNOS (*p* < 0.01), TNF-α (*p* < 0.05) and IL-1β (*p* < 0.001) and enhanced the expression of IL-10 (*p* < 0.05) (Figure 6C–E).

These results indicate that D-Exo, superior to ND-Exo, attenuates neuroinflammation by shifting microglia from a pro-inflammatory M1 state toward an anti-inflammatory M2 phenotype.

### 3.7. Prophylactic D-Exo Intervention Alleviates Cognitive Decline of HNF Mice

**Figure 7:**
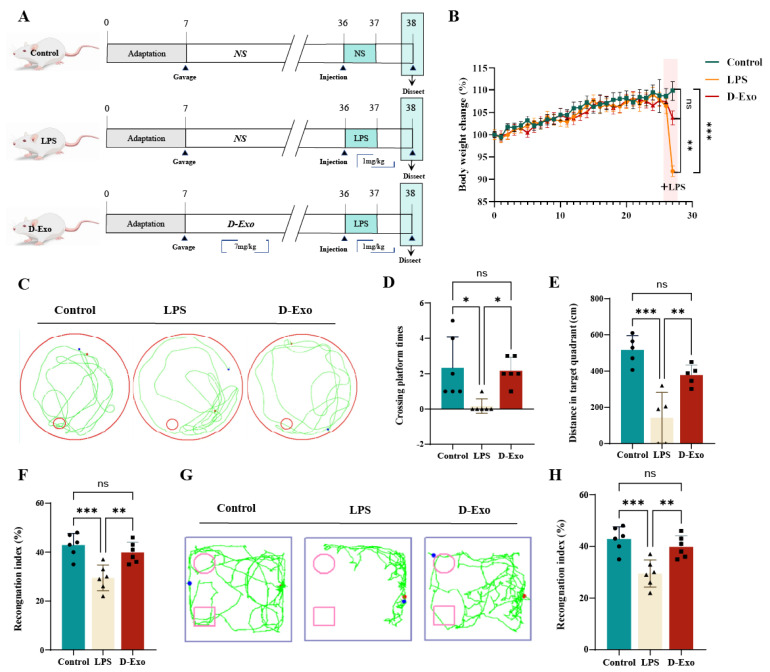
D-Exo supplementation improves the HNF mice. (**A**) Experimental strategy of mice. (**B**) Rate of body weight change in mice. (n = 8) (**C**) Representative trajectory plots from the MWM test. (**D**) Platform crossings per group (60 s). (**E**) Target quadrant time (60 s). (**F**) Distance in target quadrant (60 s). (**G**) Performance in NOR test (**H**) Recognition index of each group. Data are presented as mean ± SEM (*n* = 6 per group). Statistically significant differences were indicated: * *p* < 0.05, ** *p* < 0.01, *** *p* < 0.001, ns > 0.05.

D-Exo pretreatment for four weeks significantly attenuated the LPS-induced body weight loss in mice (*p* < 0.05) (Figure 7A,B), demonstrating its protective effect against systemic inflammation. Spatial learning and memory were assessed using the MWM (Figure 7C–F). LPS-treated mice exhibited significant impairments, with fewer platform crossings (*p* < 0.05), less time (*p* < 0.01) and shorter distance in the target quadrant (*p* < 0.001). In contrast, D-Exo administration significantly ameliorated these deficits, increasing platform crossings (*p* < 0.05), time in the target quadrant (*p* < 0.05), and swimming distance (*p* < 0.01) compared to the LPS group. These cognitive benefits were further supported by the NOR test, where D-Exo pretreatment led to a significantly higher recognition index (*p* < 0.001) (Figure 7G,H).

Together, these results demonstrate that D-Exo effectively alleviates LPS-induced cognitive decline.

### 3.8. Prophylactic D-Exo Alleviates LPS-Induced Inflammatory Responses

**Figure 8:**
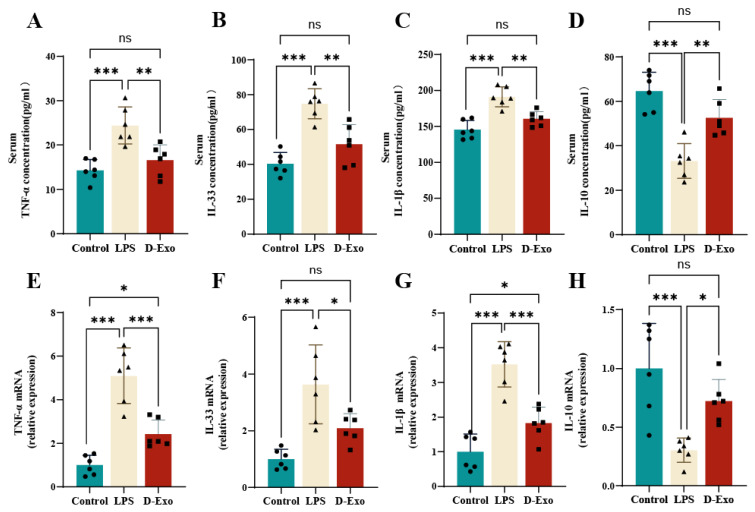
Effect of D-Exo on proinflammatory and anti-inflammatory cytokine level. (**A**–**D**) TNF-α, IL-33, IL-1β, and IL-10 level in serum by ELISA. (**E**–**H**) TNF-α, IL-33, IL-1β, and IL-10 mRNA expression in hippocampus by RT-PCR. Data are presented as mean ± SEM (*n* = 6 per group). Statistically significant differences were indicated: * *p* < 0.05, ** *p* < 0.01, *** *p* < 0.001, ns > 0.05.

To better understand D-Exo effects on serum inflammatory response, we analyzed TNF-α, IL-33, IL-1β and IL-10 level. Compared with LPS group, D-Exo significantly decreased TNF-α (*p* < 0.001), IL-33 (*p* < 0.01) and IL-1β (*p* < 0.01) level, while significantly increased IL-10 levels (*p* < 0.05) (Figure 8A–D).

The expression of inflammatory markers in hippocampal tissue was analyzed by RT-PCR. LPS challenge significantly elevated pro-inflammatory cytokines (TNF-α, IL-33, IL-1β) and suppressed the anti-inflammatory cytokine IL-10. In contrast, D-Exo effectively reversed this profile, significantly reduced TNF-α (*p* < 0.001), IL-33 (*p* < 0.01) and IL-1β (*p* < 0.01) expression, and significantly increased IL-10 (*p* < 0.05) expression (Figure 8E–H). These results confirm the potent anti-inflammatory activity of D-Exo in vivo.

### 3.9. Prophylactic D-Exo Intervention Alleviates Hippocampus Damage and the Expression of Tau/A_β1-42_ Protein in of HNF Mice

**Figure 9:**
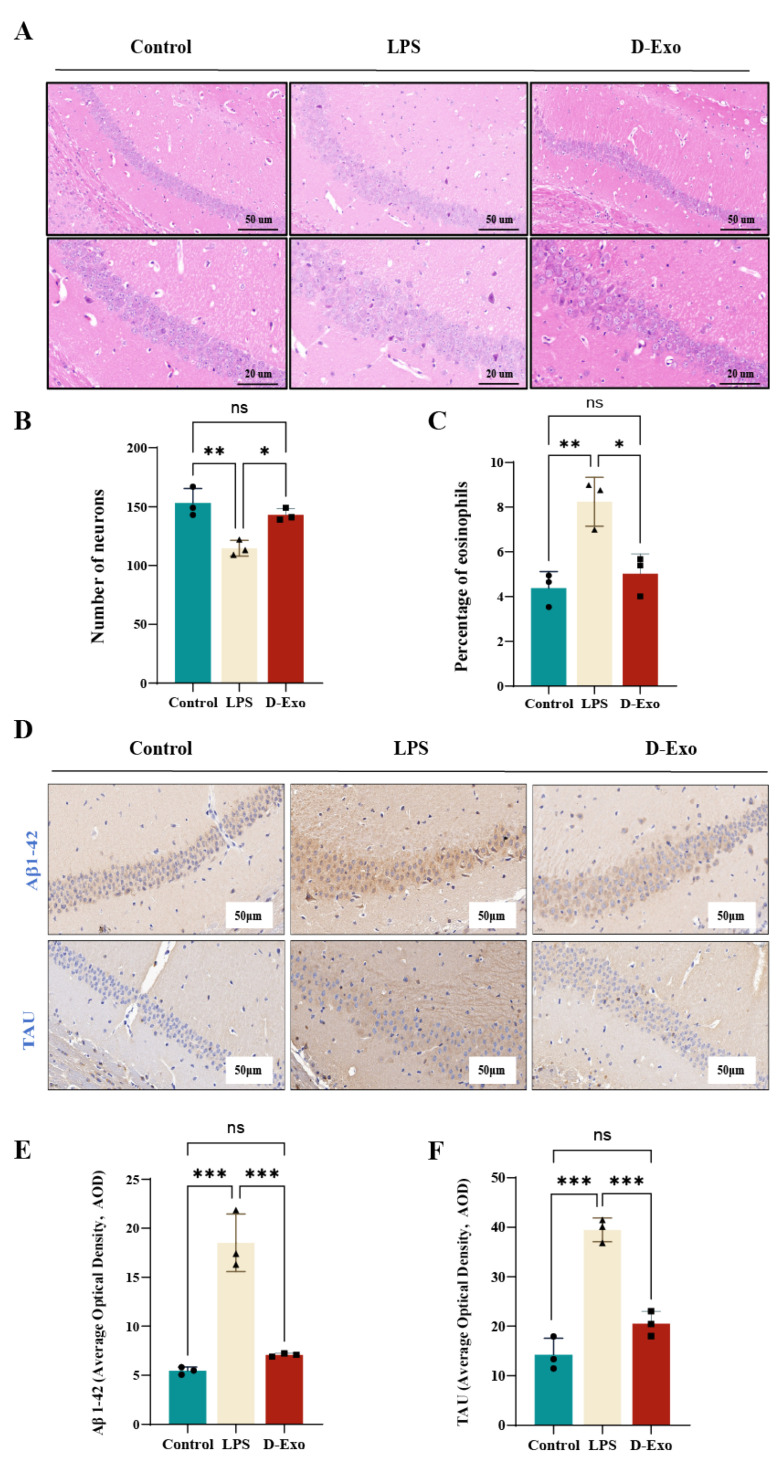
D-Exo protects against LPS-induced hippocampal damage and pathological accumulation of Tau and Aβ_1-42_. (**A**) Representative image of the hippocampal pathological morphology by HE staining (50-20 μm). (**B**) The number of neurons in the CA1 region in the hippocampus. (**C**) Percentage of eosinophils in the CA1 region in the hippocampus. (**D**) Representative images of Aβ_1-42_ and Tau proteins by IHC. (**E**) Quantification of Aβ_1-42_ protein immunohistochemical staining using AOD values. (**F**) Quantification of Tau protein immunohistochemical staining using AOD values. Data are presented as mean ± SEM (*n*= 3 per group). Statistically significant differences were indicated: * *p* < 0.05, ** *p* < 0.01, *** *p* < 0.001, ns > 0.05.

HE of the hippocampus is presented in Figure 9A. Neurons in the LPS group exhibited loose arrangement, enlarged intercellular gaps, and heterogeneous morphology, with evidence of nuclear condensation, lysis, or loss. Neuronal density (*p* < 0.01) and the proportion of eosinophils (*p* < 0.05) were significantly reduced. Compared with LPS group, the morphology of neurons in D-Exo group was significantly improved and close to that of the control group. The number of neurons (*p* < 0.05) and the percentage of eosinophils (*p* < 0.05) were significantly increased (Figure 9A–C). It indicates that D-Exo can effectively improve the neuronal impairment in mice induced by HNF.

IHC analysis of Tau and Aβ_1-42_ in the hippocampal CA1 region is shown in Figure 9D–F. Within the hippocampus, Aβ_1-42_ was visualized as widespread, cytoplasmic brown-yellow precipitates. D-Exo significantly reduced the average optical density of Aβ_1-42_ (*p* < 0.001) and Tau (*p* < 0.001) compared to the LPS group. These results indicate that D-Exo contributes to the preservation of neuronal cytoskeletal integrity and mitigates LPS-induced pathological protein accumulation.

### 3.10. Prophylactic D-Exo Intervention Enhances the Expressions of BDNF and MAP2 Protein in the Hippocampus of HNF Mice

**Figure 10:**
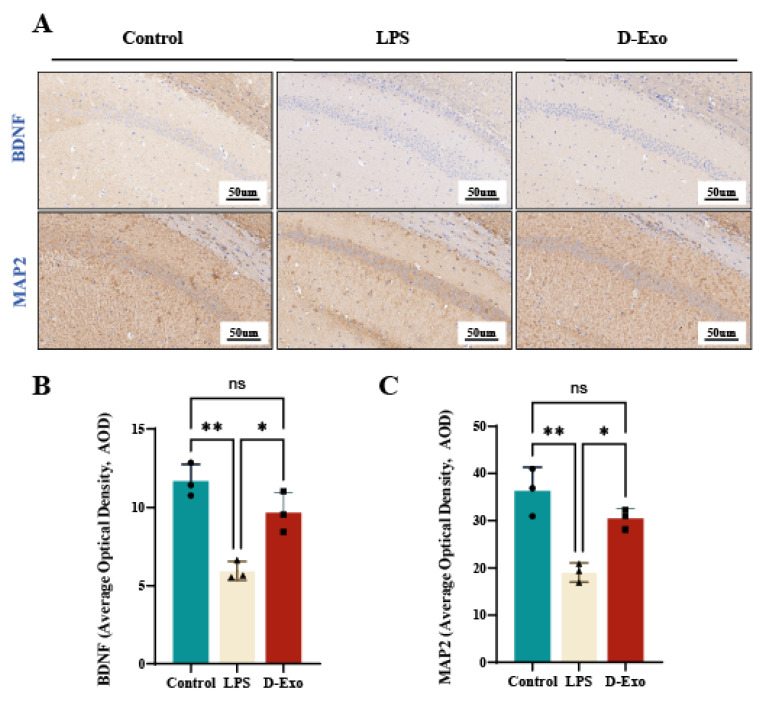
D-Exo supplementation alters the expression of BDNF and MAP2 in the hippocampus of HNF mice. (**A**) Representative image of BDNF and MAP2 proteins by IHC. (**B**,**C**) Quantification of BDNF and MAP2 protein immunohistochemical staining using AOD Values. Data are presented as mean ± SEM (n = 3 per group). Statistically significant differences were indicated: * *p* < 0.05, ** *p* < 0.01, *** *p* < 0.001, ns > 0.05.

Assessed by IHC in the CA1 hippocampus (Figure 10A), BDNF and MAP2 expression was elevated in control mice, manifested as extensive brown-yellow cytoplasmic staining. Compared with the LPS group, D-Exo group significantly increased the brown-yellow sediment average optical density of BDNF and MAP2 (*p* < 0.01) (Figure 10B,C). the IHC results suggested that D-Exo could potentially contribute to maintain synaptic plasticity via enhancing the expression of BDNF and MAP2.

### 3.11. Prophylactic D-Exo Attenuate HNF via Regulating AMPK and Glial Activatio

**Figure 11:**
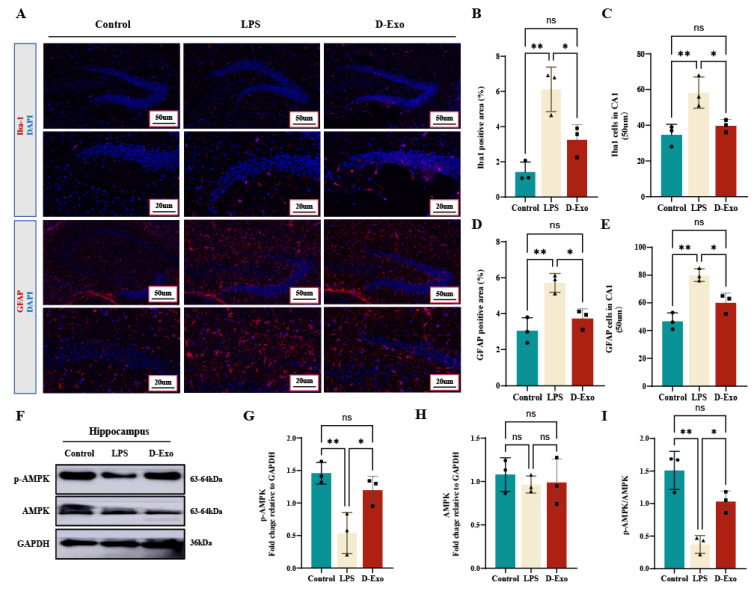
D-Exo attenuate LPS-induced HNF by modulating Microglial/Astrocytic activation and AMPK phosphorylation. (**A**) Immunofluorescence staining of Iba-1 and GFAP in hippocampus. (**B**,**C**) Quantification of data from Iba-1. (**D**,**E**) Quantification of data from GFAP. (**F**) The p-AMPK, AMPK protein with representative image by WB. (**G**–**I**) Levels of AMPK/GAPDH, p-AMPK/GAPDH, and p-AMPK/AMPK in the mice hippocampus. Data are presented as mean ± SEM (n = 3 per group). Statistically significant differences were indicated: * *p* < 0.05, ** *p* < 0.01, *** *p* < 0.001, ns > 0.05.

To evaluate the in vivo anti-inflammatory effects of D-Exo, its impact on LPS-induced glial cell activation was assessed. IF analysis revealed that LPS challenge significantly increased the Iba-1-positive area and cell count in the hippocampal CA1 region, indicative of microgliosis (Figure 11A–C). D-Exo pretreatment markedly attenuated this increase (*p* < 0.05). Similarly, D-Exo also significantly reduced the LPS-induced elevation in GFAP-positive signals, demonstrating suppression of astrogliosis (*p* < 0.05) (Figure 11A,D,E). Thus, these findings demonstrate that D-Exo ameliorates LPS-induced activation of both microglia and astrocytes in mice.

WB analysis showed that LPS treatment significantly decreased the levels of phosphorylated AMPK and the p-AMPK/AMPK ratio in the hippocampus (*p* < 0.01), while total AMPK remained unchanged (*p >* 0.05) (Figure 11F–I). D-Exo administration effectively mitigated this LPS-induced reduction in AMPK phosphorylation. These results suggest that the modulation of the AMPK signaling pathway, particularly the restoration of AMPK phosphorylation, may underlie the neuroprotective effects of D-Exo against hippocampal neuroinflammation and cognitive decline.

## 4. Discussion

HNF is a pivotal contributor to cognitive decline [35]. However, specific preventive strategies for cognitive decline and HNF remain unclear, particularly regarding the mechanisms by which milk exosomes alleviate HNF and other neurodegenerative processes. Our study not only corroborates the protective effects of milk exosomes [36,37,38] but also provides novel mechanistic insights by demonstrating that D-Exo exhibit superior bioactivity. This enhanced potency is linked to a distinct proteomic signature, which facilitates the attenuation of neuroinflammation and associated tau pathology through the modulation of microglial polarization and activation of the AMPK signaling pathway.

A key finding of this work is the differential proteomic profile identified in D-Exo compared to ND-Exo. Specifically, proteins including SIL1, FN1, NUCB2, and FGG were significantly enriched, whereas LPL and SERPINB1 were markedly depleted in D-Exo. This compositional distinction likely forms the molecular basis for its enhanced functional efficacy compared to ND-Exo. Research has indicated that SIL1 inhibits amyloidogenic APP processing and reduces Aβ_1-42_ pathology [39,40], while loss-of-function variants in FN1 attenuate pathological protein deposition at the BBB and enhance toxic aggregate clearance [41,42]. NUCB2 exerts neuroprotective effects in cerebral ischemia models; its active form, Nesfatin-1, demonstrates anti-inflammatory and anti-apoptotic properties by inhibiting astrocyte activation and regulating apoptotic protein balance [43,44,45]. Downregulation of LPL, a key enzyme in lipid metabolism [46], reduces pro-inflammatory lipid supply and thereby attenuates microglial overactivation [47]. Similarly, decreased expression of the serine protease inhibitor SERPINB1 may alleviate its suppression of phagocytosis and lysosomal function, promoting the clearance of harmful protein aggregates [48,49]. In parallel, although SIL1 and NUCB2 did not show direct interactions within this inflammation-focused network, literature indicates their protective roles are mediated via maintaining endoplasmic reticulum (ER) homeostasis and transmitting anti-inflammatory signals, respectively [40,50,51,52]. Collectively, these findings suggest that D-Exo likely alleviates neuroinflammation and cognitive decline through a coordinated, multi-level, and multi-mechanistic interplay.

Bioinformatics analysis further revealed a significant enrichment of these differentially expressed proteins in the AMPK signaling pathway. AMPK functions as a key energy sensor and a primary regulator of cellular metabolism [53,54], and its activation has been shown to suppress neuroinflammation by shifting microglial polarization from the M1 to the M2 phenotypeoften, via downstream inhibition of NF-κB signaling [55,56]. We propose that D-Exo delivers specific protein cargoes, such as FN1, FGG and others within the identified interactome, which contribute to AMPK pathway activation. This activation triggers an anti-inflammatory cascade that curbs pro-inflammatory cytokine release and promotes a microglial phenotypic shift, ultimately breaking the cycle of neuroinflammation and associated neuronal damage.

Microglial activation is a crucial aspect of HNF [57], where activated cells perpetuate inflammation and release neurotoxic mediators that directly contribute to hippocampal damage [58,59]. Based on these findings, our preliminary experiments focused on LPS-induced BV2 microglial cells models to determine whether D-Exo possesses neuroprotective and antioxidant properties. Our results demonstrate that D-Exo exhibits stronger anti-inflammatory and antioxidant activities compared to ND-Exo, consistent with its distinct proteomic profile. Furthermore, D-Exo shifts LPS-induced microglia from the pro-inflammatory M1 phenotype toward the anti-inflammatory M2 phenotype, as evidenced by downregulation of M1 markers (CD86, iNOS) and upregulation of M2 markers (CD206). This modulation of microglial polarization represents a critical mechanism through which D-Exo exerts its neuroprotective effects, aligning with strategies that aim to rebalance microglial function to resolve inflammation.

Consistent with the in vitro observations, our in vivo results confirm the preventive potential of D-Exo administration. We induced HNF and cognitive decline in mice via continuous intraperitoneal injection of low-dose LPS (1 mg/kg), establishing a consistent experimental framework [60,61]. In the LPS-induced mice model of HNF and cognitive decline, D-Exo pretreatment significantly ameliorated systemic and central inflammatory responses. This was evidenced by reduced levels of pro-inflammatory cytokines (TNF-α, IL-1β, IL-33), increased anti-inflammatory IL-10, and marked attenuation of microgliosis and astrogliosis (reduced Iba-1 and GFAP). These changes alleviated HNF and were associated with improved synaptic plasticity and preserved neuronal structure, ultimately attenuating LPS-induced learning and memory deficits. Further analysis demonstrated that D-Exo significantly reduced abnormal tau hyperphosphorylation and Aβ_1-42_ accumulation, two key pathological processes associated with neurodegenerative progression. Furthermore, the observed upregulation of hippocampal MAP2 and a trend towards increased BDNF expression suggest that D-Exo may also support synaptic plasticity and neuronal resilience, which are essential for cognitive function [62,63].

## 5. Conclusions

In conclusion, our study elucidates a multi-faceted mechanism by which desert milk exosomes confer neuroprotection against LPS-induced hippocampal neuroinflammation and cognitive decline. The superior properties of D-Exo are attributed to its unique proteome, which is enriched in proteins associated with the AMPK signaling pathway. Acting through this pathway, D-Exo promotes anti-inflammatory microglial polarization, inhibits neuroinflammation and oxidative stress, and mitigates the pathology associated with Aβ_1-42_ and hyperphosphorylated Tau accumulation. These findings significantly advance our understanding of how milk-derived exosomes, particularly from specialized sources, can influence brain health. They highlight D-Exo not merely as a general anti-inflammatory agent but as a targeted modulator of specific neuroimmune and metabolic pathways. These findings elucidate the mechanisms of milk exosomes and suggest their potential in preventing neuroinflammation-related diseases.

## Data Availability

We permit unrestricted use, distribution, and reproduction in any medium, provided the original work is properly cited. The data presented in this study are available in the ProteomeXchange Consortium via the iProX partner repository at https://proteomecentral.proteomexchange.org, accessed on 11 December 2025, with the dataset identifier PXD071863.

## Supplementary Materials

The following supporting information can be downloaded at: https://www.mdpi.com/article/10.3390/nu18020315/s1, Table S1: The top 20 highest expressed proteins in D-Exo and ND-Exo.

## Abbreviations

The following abbreviations are used in this manuscript:

HNF	Hippocampal Neuroinflammation
AD	Alzheimer’s disease
LPS	Lipopolysaccharide
A*β*_1-42_	Amyloid-beta 1-42
NFTs	Neurofibrillary Tangles
BDNF	Brain-derived Neurotrophic Factor
MAP2	Microtubule-associated Protein 2
IBA-1	Ionized calcium-binding adaptor molecule 1
TNF-α	Tumor necrosis factor-α
IL-33	Interleukin-33
IL-1β	Interleukin-1β
iNOS	Increased inducible NO synthase
BBB	Blood-Brain Barrier
D-Exo	Desert milk exosomes
ND-Exo	Non-desert milk exosomes
BCA	Bicinchoninic acid assay
TEM	Transmission electron microscopy
NTA	Nanoparticle tracking analysis
FC	Flow Cytometry
WB	Western blot
SGF	Simulated Gastric Fluid
LC-MS/MS	Liquid chromatography tandem mass spectrometry
FASP	Filter-aided sample preparation
DDA	Data Dependent Acquisition
PMSF	Phenylmethanesulfonyl fluoride
SDS-PAGE	Sodium dodecyl sulfate-polyacrylamide gel electrophoresis
MWM	Morris Water Maze
NOR	Novel Object Recognition Experiment
TA	Time to explore old object A
TC	Time to explore old object C
HE	Hematoxylin and eosin staining
CA1	Cornu Ammonis 1
AOD	Average optical density
qRT-PCR	Quantitative Real-Time Polymerase Chain Reaction
IL-4	Interleukin-4
FBS	Fetal Bovine Serum
ROS	Reactive Oxygen Species
DMEM	Dulbecco’s Modified Eagle Medium
P/S	Penicillin-streptomycin
DCFH-DA	2′,7′-Dichlorofluorescin diacetate
SEM	Standard error of the mean
ANOVA	One-way analysis of variance
PDI	Poly Dispersity Index
PPI	Protein-Protein Interaction
ER	Endoplasmic Reticulum
SIL1	SIL1 Nucleotide Exchange Factor
FN1	Fibronectin 1
NUCB2	Nucleobindin-2
FGG	Fibrinogen gamma chain

## Appendix A

**Table A1:** Primer sequence.

Gene	Primer Sequences
CD86	F:CTGCACGTCTAAGCAAGGTC
	R:CAG AACACACACAACGGTCA
CD206	F:ACCCAAGGGCTCTTCTAA
	R:TGGCCTCTTGAGGTATGT
iNOS	F:GGACCCAGTGCCCTGCTTT
	R:CACCAAGCTCATGCGGCCT
TNF-*α*	F:ACTACCTCAACCGTTCCA
	R:GAGCTTCCCAGATCACAG
IL-1β	F:AGGAGCACCTCGGTATCA
	R: GTATTGCCATCAGCGTCC
IL-33	F:CTGTTAGTTTTGTTTTGGA
	R:GTAGTAGCACCTGGTCTTG
IL-10	F:TGTGTGTTGGCTGAATTGT
	R:CTGCTCCTGGTGAGTCCTT
*β*-actin	F:ATGGTCACGCACGATTTCCC
	R:GAGACCTTCAACACCCCAGC
