# Supplementary material for: Neuroprotective Effects of Desert Milk Exosomes in LPS-Induced Cognitive Decline: Role of Microglial M2 Polarization and AMPK Signaling
Source: Nutrients. 2026 Jan 19;18(2):315. doi: 10.3390/nu18020315 (PMC12845514; doi:10.3390/nu18020315)
Supplement: Supplementary file 1 [file nutrients-18-00315-s001.zip › nutrients-4070562-supplementary.pdf]

Supporting information

Table S1 The top 20 highest expressed proteins in D-Exo and ND-Exo

Description	D-Exo	ND-Exo	Log2(FC)	*P*-value
SIL1	27.29756667	18.4888	8.80875	0.00193313
FN1	24.0091	17.4847	6.52441	0.000168858
NUCB2	26.72373333	20.829	5.89474	5.51E-05
FGG	27.20753333	21.80163333	5.40591	0.00299676
COL14A1	24.16613333	18.91945	5.2467	0.0024366
AZGP1	25.6098	20.41743333	5.19238	0.00011915
RNASE4	27.5839	22.63633333	4.94757	4.15E-06
ANG	30.02313333	25.1679	4.85523	0.000233194
SCGB2A2	27.2201	23.20286667	4.01723	5.51E-05
NPC2	24.94533333	21.24616667	3.69913	0.000724176
QSOX1	25.38483333	21.71423333	3.67059	0.000378289
cathelicidin-1	25.722	22.09753333	3.62443	0.000233194
LPO	27.37856667	24.3744	3.00415	0.000306312
SCGB1D	28.90723333	25.91076667	2.99649	0.00011915
RNASE1	25.46173333	23.21385	2.2479	0.0220799
SELENOF	22.88196667	20.6342	2.24774	0.00317967
HP	26.9479	24.83733333	2.11054	4.67E-05
UBR4	25.01953333	23.0149	2.00464	0.0376171
